# On the Value of the Chinese Pre-Qin Confucian Thought of “Harmony” for Modern Public Mental Health

**DOI:** 10.3389/fpsyg.2022.870828

**Published:** 2022-06-02

**Authors:** Yingying Li, Huaqian Cui

**Affiliations:** ^1^School of Foreign Language Studies, Wenzhou Medical University, Wenzhou, China; ^2^Health and Bioethics Research Center, Wenzhou Medical University, Wenzhou, China

**Keywords:** pre-Qin Confucianism, balanced harmony, external harmony, internal harmony, modern public mental health

## Abstract

The Chinese pre-Qin Confucianism puts forward the concept of “Harmony,” covering “Balanced Harmony,” “External Harmony,” and “Internal Harmony,” etc. “Balanced Harmony” refers to the harmonious state of balance at both ends. “External Harmony” indicates the harmonious relationship with others, with society and with nature. “Internal Harmony” reveals the harmonious state within oneself between body and mind. “Balanced Harmony” is the ideal pursuit of Harmony, “External Harmony” and “Internal Harmony” are the two basic contents of Harmony, the trinity of which constitute a systematic concept of valuing “Harmony.” The golden mean is the fundamental criterion for achieving “Balanced Harmony,” while “Benevolent people love others” and “Seeking from the heart” are the two basic requirements for achieving “External Harmony” and “Internal Harmony” specifically, and together they constitute a systematic methodology of valuing “Harmony.” As one of the backbones of traditional Chinese culture, the Chinese pre-Qin Confucian thought of “Harmony” not only has had a far-reaching impact on the temperament, mental state, cognitive style, and moral character of the Chinese nation in history but also provides insights for public mental health in modern times globally, containing a great deal of useful information for mental health issues from which Western public mental health could profit.

## Introduction

Pre-Qin period refers to China’s historical period from the Paleolithic Age to the establishment of the Qin Dynasty (221 BC). Pre-Qin Confucianism is founded by Confucius (551–479 BC) in the pre-Qin period and is one of the 100 schools of thought represented by Mencius and Xunzi, etc. The pre-Qin Confucianism attaches great importance to the significant position and positive functions of “Harmony,” holding that “The change of the way of heaven makes all things get their life right. All things are endowed with their qualities and characteristics. Through the coordinated and harmonious interaction of all things, the Grand Harmony is preserved” (Huang and Zhang, 2018, p.74). It is implied that all things must be in harmonious relation with nature to grow, and through mutual coordination, they maintain the highest harmonious state and achieve great prosperity. Xunzi maintains that “The growth of all creatures depends on Harmony” (Wang, 2012, p. 302), stressing that Harmony is a necessary condition for the prosperity and growth of life and vitality. The concept of Harmony, such as “Harmony in diversity” (Zhu, 2016, p. 134), “Harmony is precious” (Zhu, 2016, p. 45), constitutes the basic contents of valuing Harmony, covering “Balanced Harmony,” “External Harmony,” and “Internal Harmony.” The golden mean is the fundamental criterion for achieving “Balanced Harmony,” while “Benevolent people love others,” and “Seeking from the heart” are the two basic requirements for realizing “External Harmony” and “Internal Harmony” specifically, and together they constitute a systematic methodology of valuing “Harmony,” which meets the needs of the pluralistic integration of the Chinese Nation and “may have been responsible for holding the country together throughout its long history” (Wong, 2016). After Dong Zhongshu (179–104 BC), the successor and developer of Confucianism in the Western Han Dynasty, Confucianism developed into the “Famous School of thought” which had the greatest influence on the Chinese people. Some frequently-quoted proverbs related to Harmony, such as “Harmony begets wealth” (Lao, 2008), “Harmony brings all nations together” (Wang, 2012), “A harmonious family prospers all things” (Wu, 2011), and “Harmony is the key to a family” (Zhang, 2013), have spread far and wide in China and have helped to shape the personalities and moral characteristics of the Chinese people.

The term “Mental health” is a modern psychology category originating from the west, which is, in some way, in agreement with the philosophical ideas held by Chinese pre-Qin Confucianism in terms of “Harmony.” In 1948, on the first International Congress of Mental Health, mental health was defined as follows: “Mental health is regarded as a condition which permits the optimal development, physical, intellectual, and emotional, of the individual, so far as this is compatible with that of other individuals” (Bertolote, 2008). The Committee on Ethical Issues of the European Psychiatric Association proposes the definition of mental health, holding that it is a dynamic state of internal equilibrium which enables individuals to use their abilities in Harmony with universal values of society and harmonious relationship between body and mind (Galderisi et al., 2015). It can be seen from the above discussions that “mental health” is essentially a “harmonious” state of individual psychology, with “Harmony” as the goal, and equilibrium within oneself and with others and the environment as the foundation. It is considered that mental health largely depends on how effectively one deals with internal tension and external conflicts, and the key to maintaining mental health lies in managing internal tension and external conflicts in a moderate way so as to stay in a good or harmless psychological state (Huang, 2020). Mental health, in the sense of modern psychology, to a large extent, is reflected in a state of Harmony, mainly manifested in the “Internal Harmony” within oneself, and the “External Harmony” with others, with society and with nature, which has an inherent fit with the pre-Qin Confucian thought of valuing “Harmony.”

Ancient Chinese sages, including pre-Qin Confucians, also discuss the issue of mental health. In this regard, Wang (1993, p. 7) points out, “Ancient China had rich ideas of mental health and China is one of the most ancient origins of traditional mental health. More than 2,000 years ago, well-known books such as *Dao De Jing*, *Zhuangzi*, *Analects of Confucius*, and *Huangdi’s Inner Canon of Medicine* all abound in rich elements of mental health.” Li (2018, p. 47) also points out that “The basic feature of Confucianism lies in shaping human psychology.” The pre-Qin Confucian thought of valuing “Harmony” contains rich wisdom of adjusting mental conflicts and maintaining mental harmony, which can benefit public mental health development for people in modern times worldwide.

Pre-Qin Confucian teachings provide inspiration and insight for Chinese Positive Psychology. Wong (2016) proposes that traditional Chinese cultural beliefs have great potential to influence Chinese Positive Psychology as well as mainstream psychology, making Chinese Positive Psychology show its own distinctive features. Firstly, Chinese Positive Psychology values balance and Harmony. According to Chinese Positive Psychology, balance and moderation are more valued than achieving optimal levels of individual functioning and happiness. Group Harmony is considered more important than individual success. The ideal life is to live a simple life in peace and harmony with family members and neighbors. Secondly, Chinese Positive Psychology features both “External Harmony” and “Internal Harmony.” Wong and Sproule (1984) argue that the Chinese people can hold an external and internal locus of control simultaneously. Such dialectical thinking poses a challenge to any psychological measure that depends solely on unidimensional rating scales. It is believed that Chinese Positive Psychology serves as a bridge between Confucius teaching and Western psychology.

## Achieving “Balanced Harmony” by “Following the Golden Mean”

The term “Balanced Harmony” (*zhōng hé*, 中和), deriving from *Book of Rites*, refers to an ideal state of human mind. “When joy, anger, sorrow, and happiness are not yet expressed as a response to other things, they are in a state of Balance (*zhōng*, 中). When they are expressed in words and deeds in accordance with the rites, Harmony (*hé*, 和) is achieved. Balance is the foundation while Harmony is the universal rule under heaven. If a ruler can achieve “Balanced Harmony,” both heaven and earth will be in their proper places, which will deliver common prosperity for all” (Zhu, 2016, p. 17). It can be inferred that “balance” is the foundation of stabilizing the world, while Harmony is a way of interacting with others (Key Concepts and Terms in Chinese Thought and Culture, 2022). “Balanced Harmony” is an ideal state of the human mind and the perfect equilibrium between two ends achieved by the proper application of *zhong*. The most distinctive feature of “Balanced Harmony” lies in the balance between two ends, such as the Harmony for individuals between body and mind, the Harmony between people, such as “Emperor” and “minister,” “father” and “son,” “husband” and “wife,” and “older brother” and “younger brother,” while the Harmony between man and the outside world emphasizes the balance between “man” and “heaven.” Later on, “Balanced Harmony” is introduced into the field of health and regarded as an important method for nourishing the body and mind and prolonging life. In Dong Zhongshu’s view, when one achieves “Balanced Harmony” in self-cultivation, they will enjoy longevity” (Zeng, 2009, p. 363). Similarly, Tao (2011, p. 16), a famous Chinese medical scientist in the Southern Dynasty, also regards “Balanced Harmony” as an effective way of keeping good health and prolonging life.

How to maintain “Balanced Harmony?” Pre-Qin Confucianism believes that the golden mean is the fundamental law to achieve “Balanced Harmony,” and pursuing the golden mean is the fundamental path to achieving “Balanced Harmony.” The term the golden mean (*zhōng yōng*, 中庸）), deriving from *Analects of Confucius*, refers to the standard of moderation that one should follow in dealing with others and in one’s everyday conduct, which is considered to be the highest level of virtue by Confucius and Confucian scholars (Zhu, 2016, p. 81). As Zhu Xi, the foremost Neo Confucian thinker in the Southern Song Dynasty, illustrates, moderation in one’s words and deeds and not bending one way or the other is *zhong*, and remaining unchanging is *yong*. *Zhong* is the right way in the world while *yong* is the common principle under heaven (Zhu, 2016, p. 16). Confucius regards whether one could adhere to the golden mean as an important standard to distinguish a man of virtue from a villain, stressing that neither exceeding nor falling short of the line is desirable, both of which deviate from the way of the golden mean. The doctrine of the mean requires people to effectively regulate their emotions, such as joy, anger, sadness, fear, love, disliking and liking, and to keep them in a balanced and peaceful state to remain mentally healthy.

Concerning how to follow the golden mean, pre-Qin Confucians put forward a series of specific methods, four of which are discussed as follows, namely, “holding fast the golden mean,” “hammering at two ends,” “finding truth between two sides,” and “changing with time.” First of all, one should “hold fast the golden mean” (*yun zhí jué zhōng*, 允执厥中). The term “hold fast the golden mean” originates from *Shangshu*. Confucian scholars believe that human conscious mind involves two aspects: one is the “moral heart” (*dào xîn*, 道心), which refers to being conscious of moral rules in agreement with heavenly principles, the other is the “human heart” (*rén xîn*, 人心), which is consumed with desires. The “moral heart” needs to be allowed full play so that the excessive desires of the “human heart” can be held in check. “The human heart is beset by danger, while the moral heart is subtle and elusive. Concentration of mind is required for sticking to the path of justice and uprightness” (Zhu, 2016, p. 14). This is the frequently-quoted “Sixteen-Character Theory of the Heart” proposed by pre-Qin Confucianism, which requires people to uphold the golden mean, adjust the “human heart” with the “moral heart,” and stand erect in the middle without inclining to either side to attain peace of mind (Zhu, 2016, p. 19). Mental health does not imply that there is no psychological deviation, but that in case there is psychological deviation, it can return to the state of psychological balance through self-regulation.

Secondly, in handling with difficult issues in life, to find out the golden mean, one is expected to “hammer at two ends” (*kòu qí liang duān*, 叩其两端). “Hammer at two ends” originates from *the Analects.* Confucius shares a story with Zi Han, one of his disciples, “When a man asks me questions about a subject, I have no idea of what it is. Then by asking questions about the pros and cons and hammering at both ends I get everything out of it.” Zhu (2016, p. 99) illustrates that two ends means two sides or two parties and if we look at an issue from both sides we will find out more. “Harmony” is based on the recognition of the opposite sides of the unity. If there is only one party, there is only “uniformity” and “Harmony” won’t exist at all. “Hammering at both ends” aims to require people to start from the “two ends” and look at problems comprehensively. The psychological problems of modern people are often caused by the comprehensive influence of the increase of external pressure and internal one-sided cognitive deviation. Psychological balance is not only a psychological state but also a process of psychological adjustment. Only by “hammering at both ends” and looking at problems dialectically, can we adjust cognitive bias, improve defense mechanism, strengthen psychological endurance, avoid extreme mental problems, and maintain psychological balance.

Thirdly, while “hammering at both ends,” we attempt to “find the truth between two sides” (*zhí liang yòng zhōng*, 执两用中), which originates from the *Book of Rites:* “He takes hold of the two extremes and finds the truth between two sides, and employs it in his government of the people” (Zhu, 2016, p. 18). It is implied that the ruler should uphold the golden mean of governing the nation. Because it is difficult to adhere to the doctrine of the mean, people often deviate from it. Therefore, it is necessary to “find the truth between two sides” and return things to the state of “Balanced Harmony.” However, occasionally, things might deviate from the golden mean, and when this occurs, it is a necessity to make things return to the golden mean through artificial adjustment, which is just like when a chef seasons the food to make all kinds of flavors just right; if the flavors are not enough, he will add more seasonings, and if the flavors are too strong, he will reduce seasonings or add water to dilute it. In this way, the soup will surely produce a better taste (Yang, 2006, p. 1,419). Similarly, this is how traditional Chinese medicine treats diseases and applies to psychotherapy, too. The essence of psychological balance therapy is to adjust the fulcrum of balance and make the unbalanced mentality return to balance. Supposing you are overconfident and conceited or overjoyed when you succeed, you suffer from overmuch emotional experience and you must try to reduce it. Conversely, if you lack confidence or feel inferior, or feel discouraged when you have setbacks, you need comfort and encouragement. Only by adjusting the mental state to proper moderation can people stay mentally healthy.

Fourthly, it is advisable that people deal with difficulties with flexibility and “change with time” (*yìng shí ér biàn*, 应时而变). Admittedly, Confucian scholars regard the mean as the supreme principle guiding people’s behavior. However, the criterion for determining what is appropriate or not does not remain unchanged. Pre-Qin Confucians emphasize that since things are constantly changing, only by understanding “following the mean” and “temporarily changing aspects of *Dao*” can we meet the requirements of the mean. When taking a specific action, one should always bear in mind the need to observe a change of the function of *Dao* to suit circumstances or to meet the needs of the day. Confucius proposes that a man of virtue (*jūn zi*, 君子) should seek to be in keeping with the mean at all times (Zhu, 2016, p. 17), and he is also regarded as a sage of flexibility by Mencius (Zhu, 2016, p. 180). “Such is Confucius: he went into office if he could, and withdrew, if not; he held office for long if he could, and made it short if not. Sticking to the middle course is to get close to being correct. But sticking to the middle course without flexibility is as wrong as going to extremes” (Zhu, 2016, p. 211). If you are mechanically loyal to the middle way without understanding contingency, you will eventually deviate from it (Zhu, 2016, p. 317). For example, in ancient China, when males and females were not allowed to give or receive things from hand to hand, supposing you were confronted with your drowning sister-in-law, would you offer a helpful hand to save her life? If so, that is flexibility (Zhu, 2016, p. 105). Otherwise, it would be nothing but ruthless murdering. Psychological balance is not only a good psychological state, but also a dynamic psychological adjustment process. Piaget (Wadsworth, 1989), a Swiss psychologist, believes that an individual’s cognitive schema is adapted to a new environment through a dynamic balance of assimilation and adaptation. “Changing with time” and being flexible are the requirements to maintain a dynamic psychological balance.

Modern medicine reveals that emotional imbalance, disorder and anything out of control will lead to various mental problems, such as physiological dysfunction, metabolic disorder, decline of immune function, and other hazards. As described in *Huangdi’s Inner Canon of Medicine*, anger damages the liver, overjoying hurts the heart, anxiety impairs the spleen, grief injures the lung, and fear the kidneys, etc. (Li, 2005, p. 64). It is argued that by moderating your feelings like joy or sorrow, your life can be prolonged (Gong and Lu, 2014, p. 226) since emotions are social and emerge from dynamic interactions between individuals and their social environment (Cho et al., 2018). Peng and Nisbett (1999) point out that Chinese thinking is dialectical rather than logical. The fundamental path of “following the golden mean” helps guide people to correctly understand and handle the dialectical relationship of the “two ends” between man and me, material and spirit, gain and loss, etc., remain open-minded and tolerant, adjust the fast-paced stressful life and try to maintain a good mentality for “Balanced Harmony,” to enjoy happiness in the process of pursuing progress.

## Maintaining “External Harmony” by Means of “A Benevolent Person Loving Others”

“External Harmony” (*wài hé*, 外和) indicates good relationship with other people and the outside world as well, which helps make people stay happy and healthy. This concept of “External Harmony” is embodied in the remarks and teachings made by Confucius and Confucian scholars. Firstly, they attach great importance to the Harmony between people and design moral norms such as “benevolence” and “propriety” to regulate interpersonal relations. Confucius stresses that “Harmony is the most important” (Zhu, 2016, p. 45). Mencius believes that “Favorable weather conditions are not as valuable as favorable geographic conditions, and favorable geographic conditions are not as valuable as Harmony among people” (Zhu, 2016, p. 217). In addition, the pre-Qin Confucianism also values the Harmony between man and nature. Mencius emphasizes knowing Heaven and standing in awe of Heaven. Similar discussions can be found in *Book of Rites*, for example, “All things grow together without harming each other” (Zhu, 2016, p. 370), “If one is able to assist the transforming and nourishing powers of Heaven and Earth, he or she may be equally important as Heaven and Earth” (Zhu, 2016, p. 32). Confucians have faith in this ultimate Harmony among the things in the world, maintaining that all the different things harmonize even as they constantly change (Li, 2006).

People always live in a certain environment, and constantly exchange matter and energy with the external world. External harmonious relationship is an important measure of modern mental health. American psychologists Maslow and Mittelman regard “being able to maintain a good interpersonal relationship” and “keeping in touch with the environment” as two important mental health standards (Fan and Fei, 2006, p. 151). Cai Zhuoji, a Chinese psychologist, also regards good interpersonal Harmony and adapting to the environment and dealing with setbacks as two important standards of mental health for Chinese people (Mei, 2011, p. 5). Modern medical and health technology has proved that environmental pollution will endanger people’s physical and mental health. This has an internal coincidence with the “External Harmony” view of pre-Qin Confucianism.

To maintain “External Harmony,” pre-Qin Confucianism proposes that one must have a loving heart and be benevolent. The concept “A benevolent person loves others” originates from the *Analects of Confucius*, “Fan Chi asks about benevolence, and the Master replies, “Love your fellow men” “(Zhu, 2016, p. 126). Confucianism regards benevolence (*rén*, 仁) as the highest moral value. With “loving people” as the basic connotation, the concept of “Benevolent people love others” follows the way of loyalty and forgiveness and the principle of “love has differences,” from inside to outside, from near to far, from loving relatives to loving others, society, and all things in the world. As Zhao (2009) puts it, “Ethical transposition is held to develop in an ascending order, from families to states and then to “all-under-heaven”.”

Regarding benevolence, above all, one must love one’s family members. The love for the close kinsfolk is regarded as human goodness. As an ideal state of family relations, the harmonious coexistence in family life matters on individual mental health (Zhan and Wang, 2021). Confucius stresses, “A young man must behave well to his parents and his elders” (Zhu, 2016, p. 42), pointing out that “A man of virtue devotes his efforts to the roots, for once the roots are established, *Dao* will grow therefrom. Being good as a son and obedient as a younger brother is, perhaps, the root of a man’s character” (Yang, 2008). Confucius’s disciples are requested to be filial to their parents when they are at home and respect their elder brothers when they go out (Zhu, 2016, p. 43). Since in ancient China transportation was underdeveloped, it makes sense that Confucius proposes that “while your parents are still alive, you’d better not travel too far afield. If you do travel, you should always let them know where you are going” (Zhu, 2016, p. 64). Moreover, filial piety requires people to not only care about their parents’ necessities of life, such as residence, maintenance, disease, funeral, etc., but, more importantly, always respect their parents from the bottom of their hearts. Even when your parents do something wrong, you’d better dissuade them from doing wrong most gently. Even if your advice is ignored, you should not become disobedient but should remain reverent (Zhu, 2016, p. 49). In serving parents, only when one remains respectful all the time can he be regarded as a good son. In addition, only showing love to parents is not enough, a man must be earnest and genial amongst brothers before he deserves to be called a man of virtue (Zhu, 2016, p. 134). Mencius points out that no benevolent men would ever leave his parents untended, nor would a righteous man neglect his sovereign (Zhu, 2016, p. 183). Xunzi makes similar remarks, “The essence of benevolence is to serve the parents (Zhu, 2016, p. 257), and “Humane behavior is the manifestation of love, and thus it is expressed in one’s treatment of relatives” (Zhu, 2016, p. 475).” Benevolence is the characteristic element of humanity and the great exercise of it is in loving relatives (Zhu, 2016, p. 25).

Only loving one’s relatives is far from enough. From Confucius’s perspective, one should extend broad love to all. The pre-Qin Confucianism extends the spirit of benevolence from one’s family members to all people of the society. Confucius holds that a benevolent man helps others to take their stand in that he himself wishes to take his stand, and gets others there in that he himself wishes to get there (Zhu, 2016, p. 81). He advocates friendliness, love, and understanding among people and advises people not to impose on others what they themselves do not desire (Zhu, 2016, p. 120). Besides, he urges rulers to keep expenditure under proper regulation, love their fellow men and employ the labor of the common people in the right seasons (Zhu, 2016, p. 42). That is, being frugal and caring for the people, giving them time to recuperate. It is proposed that all the people in the world are as close as brothers (Zhu, 2016, p. 122). In Mencius’s view, “A man of virtue keeps in heart benevolence. Those who love others are constantly loved by others. Those who respect others are constantly respected by others” (Zhu, 2016, p. 267). The love between people, just like the action and reaction in physics, is mutual. Only by respecting and loving each other can we live in Harmony. Mencius advocates that people “Respect your own elders and extend such respect to those of others; cherish your own young and extend such cherishment to those of others” (Zhu, 2016, p. 190). Mencius synthesizes and upgrades this notion into a theory to be applied to the governance of a country, holding that when a sovereign practices commiserative governance with a merciful heart, he will find it easy to rule the kingdom (Zhu, 2016, p. 213–214). Xunzi also requires the ruler to love and benefit his people (Wang, 2012, p. 230).

Positive Psychology believes that social support, which refers to the influence obtained by a person through social contact that can reduce psychological stress, relieve mental tension, and improve social adaptability (Li, 1998, p. 67), is closely related to subjective well-being. Social contact means interpersonal relationship and is covered by “External Harmony.” The concept of social support was first introduced into psychiatric research, mainly to explore the impact of social support on mental health. Research shows that positive social support comes from the encouragement and comfort of relatives, friends, and others. It can enhance people’s subjective well-being, positive emotional experience and empathy consciousness, give people psychological comfort, stimulate people’s psychological potential, and help maintain people’s psychological health. The pre-Qin Confucianism’s proposition of building a harmonious family and social relationship through loving relatives and extending love to all is similar to the view that the positive social support of Positive Psychology is conducive to enhancing happiness.

Furthermore, the pre-Qin Confucianism extends the spirit of benevolence to love all things in the world. Although Confucius does not explicitly put forward the proposition of benevolence for all things, his love for animals and nature can be revealed from the following remarks, “The master uses a fishing line, but not a big net. He does not shoot at roosting birds” (Zhu, 2016, p. 89). It can be inferred that a large net will catch more fish, which will cause harm to fish and nature; when birds return to their nests, they need to inhabit and breed, both of which reflect Confucius’s love for animals and nature with restraint and moderation. Another example is that when the dog in Confucius’s family died, he instructed Zi Gong, one of his disciples, to wrap up the dead body and bury it, showing his love for animals. Mencius puts forward the idea of benevolence and love for all things, saying “Men of virtue love and care for their loved ones, they are therefore kind to other people. When they are kind to people, they treasure everything on earth” (Zhu, 2016, p. 322). Later on, Dong Zhongshu emphasizes that even birds, beasts, and insects must be loved, saying, “Sincerely care for the people, down to the birds, beasts, and insects. If people do not care for the animals, how can they be called “benevolence” (Su, 1992, p. 251)? Zhang Zai, a thinker in the Northern Song Dynasty, proposes that “All people are my brothers and sisters, and all things and I are born of heaven and earth” (Zhang, 1978, p. 62). He emphasizes that all things in heaven and earth are our friends, echoing the proposition of benevolence and love of all things in pre-Qin Confucianism. Experiments in modern psychology show that intervention in the natural environment has a positive effect on controlling mental fatigue and improving the performance of inhibitory tasks (Li and Zhang, 2018, pp. 78–79). The pre-Qin Confucians advocate building a harmonious relationship between man and nature by loving all things, which is in internal agreement with the natural environment intervention proposition of modern psychology.

If people cannot maintain a harmonious relationship with others or the environment, psychological disorder might occur. It is believed that interpersonal conflicts will lead to mental problems, esp. for people in modern times (Fan and Fei, 2006). Pre-Qin Confucianism’s teaching of “Benevolent people love others,” through hierarchical and progressive benevolence, creates external harmonious relations at different levels such as family, society, and the world. It encourages people to form a positive emotional experience by exchanging and communicating with others and to improve positive self-evaluation in the process of loving others, which provides a solid external guarantee for maintaining people’s mental health. It is noticeable that the Confucian thought of “Benevolent people love others” is following the relevant propositions of Positive Psychology.

## Maintaining “Internal Harmony” by Means of “Seeking From the Heart”

“Internal Harmony” comes from “*Book of Rites, Music Book*”: “If a person has “Internal Harmony” and courtesy in appearance, people will not argue with him or her when they see his or her expressions” (Kong, 1980, p. 1,544). “Internal Harmony” refers to the inner Harmony of body and mind. Modern psychology believes that health is a state of complete physical, mental, and social well-being and not merely the absence of disease or infirmity (Liu, 1996, p. 46–48). The important standard of mental health is to keep the integrity and harmony of personality, to know oneself and accept oneself (Wu and Huang, 2007, p. 8; Mei, 2011, p. 5). It is implied that individuals should put group rules above individual needs and observe group rules first, which is, for one thing, conducive to social Harmony and stability, for another, however, prevents individual’s legitimate needs and desires from being satisfied, and their emotions from being released, which might lead to psychological problems such as anxiety, paranoia, timidity, and so on. Pre-Qin Confucianism advocates filial piety, proposing that one should obey all orders of his parents (Yang, 2010, p. 614) and not against their will (Yang, 2010, p. 329). When one sees the intimate friend of his father he cannot go forward to his father’s friend without being told to do so, not leave without being told and not talk without being questioned, which is deemed as the appropriate conduct of a filial son according to pre-Qin Confucianism (Yang, 2010, p. 5). These teachings are conducive to the stability of family order, however, it is undeniable that the authoritarian filial piety method might cause children to suffer miserably and will experience mental health problems as they grow up. To make up for this deficiency and motivate people to voluntarily abide by social norms and fulfill social responsibilities, Confucian scholars also attach great importance to giving full play to people’s inner subjectivity, and constructed methods of inward seeking and self-reflection, which provides strong support for people to adjust their mentality and eventually contributes to Internal Harmony. It can be seen that both pre-Qin Confucianism and modern psychology value the inner subjectivity of human beings.

How to keep “Internal Harmony?” Pre-Qin Confucianism believes that the fundamental way to maintain “Internal Harmony” is “seeking from the heart.” The view of “seeking from the heart” (*nèi qiú yú xîn*, 内求于心) originates from Mencius, “It is right not to express spirits about what cannot be sought from the heart. But it is wrong not to seek from the heart what cannot be expressed in words” (Zhu, 2016, p. 208). That is to say, you can seek from the heart and look for the reason whenever you are upset or say anything wrong. This discussion aims to ask people to go deep into their hearts and strengthen self-examination. It implies that you have to listen to your heart and accept what you desire. Mencius believes in the theory of original goodness of human nature, holding that the four initiators, namely, commiseration, shame, deference and a sense of right and wrong, are buds of four virtues, i.e., benevolence, righteousness, propriety, and wisdom, all of which are rooted in man’s mind (Zhu, 2016, p. 315). The end of learning is merely to help recover the lost goodness in the mind (Zhu, 2016, p. 296). To put it another way, the fundamental way of moral cultivation is to look inward for the good heart that people were born with but might have been discarded. Conversely, starting from the theory of original evil of human nature, Xunzi holds that human nature is evil and that any good in humans is acquired by conscious exertion (Wang, 2012, p. 63). He proposes to develop human subjectivity and strengthen self-cultivation to transform innate nature (Wang, 2012, p. 7). Although Mencius and Xunzi hold different views on the theory of human nature, they both focus on giving full play to people’s internal subjectivity. Modern psychology believes that any external psychological intervention and psychotherapy requires the cooperation of the object’s internal subjectivity. Psychotherapy such as Vipassana and Mindfulness aims to seek and arouse people’s internal subject potential, trigger people’s internal positive changes, cultivate positive psychology, and resolve negative ideas, which is highly consistent with the “seeking from the heart” method advanced by pre-Qin Confucianism.

To “seek from the heart,” first of all, one should always “look within and examine yourself” (nèi *zì xing*, 内自省) or conduct introspection, which originates from the *Analects of Confucius*. Confucius remarks that when you meet someone better than yourself, look within and examine your own self to become his equal, and when you meet someone not as good as you are, look within and examine yourself (Zhu, 2016, p. 64). As a way of moral cultivation, the method of looking within and examining yourself refers to the self-examination of one’s own words, deeds, and inner thoughts. Confucian scholars elaborate on this view from different perspectives. Confucius believes that through introspection, one can learn from a man of virtue and avoid bad behaviors (Zhu, 2016, p. 73). Zengzi, in particular, stresses that one should reflect many times every day on whether he or she has performed duties for others, treated others with good faith and whether he has reviewed what he has learned to see if there is any room for improvement (Zhu, 2016, p. 42). Mencius proposes that whenever you fail to achieve the desired result, you need to conduct retrospection to find out the cause (Zhu, 2016, p. 151). Mencius also shares his experience of conducting introspection, “I examine my conscience and find I am really truthful and there can’t be any greater pleasure than this” (Zhu, 2016, p. 311). Xunzi recommends the gentleman to examine himself each day while broadening his learning (Wang, 2012, p. 2). He also proposes that when having the goodness, people should firmly respect and love themselves; otherwise, people should be ashamed of themselves (Wang, 2012, p. 21). Moreover, Confucian scholars encourage people to be careful with their conduct and consciously follow morality even when they are at leisure and alone. “A man of virtue is cautious when he is not being watched by others and apprehensive when what he says is not being heard” (Zhu, 2016, p. 16). The above mentioned discussions all agree on the concept of introspection and self-discipline and aim to require people to reflect on themselves every day. Introspection has something in common with Vipassana therapy advanced by modern psychology. Vipassana therapy is to guide the subject to self-awareness and correction by means of individual experience recalling and reviewing personal emotional experience, which helps to “heal depression and anxiety full of negative thoughts, disgust, and hatred.” The main method is to guide the subject to consciously observe the precepts, train the mind, limit desires, and control impulses (Lau, 2021). Introspection is also to use the method of self-reflection to comprehend truth and to seek virtue from oneself by means of strengthening self-reflection, self-examination, self-discipline, and self-enlightenment, which is conducive to people’s development of a generous and open-minded character, and resolves various mental diseases caused by narrow-mindedness and quibbling about unimportant things.

Secondly, one is supposed to rectify one’s heart (*zhèng xîn*, 正心). The term “rectify one’s heart” means following moral principles in daily life. It is one of the eight essential principles indicating important stages in the moral cultivation from the philosophical text *The Great Learning*, the other seven being “studying things,” “acquiring knowledge,” “being sincere in thought,” “cultivating oneself,” “regulating one’s family well,” “governing the state properly,” and “bringing peace to all under heaven” (Zhu, 2016, p. 5). These eight essential principles is an inseparable whole, with “self-cultivation” as the core, while “rectify one’s heart” is the premise of self-cultivation and remains the key link connecting the preceding and the following. “Rectifying one’s heart” has something in common with mindfulness therapy. Mindfulness therapy, through breathing, yoga, meditation, and other training, helps subjects to consciously perceive their daily activities, reduce stress, manage emotions, and regulate physical and mental states. The core is to gradually guide subjects to use their inner strength to cultivate mindfulness and gain inner balance and peace. Surveys have shown that mindfulness therapy can reduce stress and have a positive impact on the quality of life and post-stroke depression (Lau, 2021). Rectifying one’s heart requires great efforts and remains a goal of self -cultivation. If one can keep in heart benevolence and proprieties as well, limit desires, remain honest and upright and cultivate the heart, an unrighteous heart can be rectified and return to the right position, which is neither exceeding nor falling short of the line. The rectified heart takes “sincerity” as the premise to make the idea sincere. If people can be “sincere” in thought, mind can be upright and rectified naturally.

“Exerting one’s heart to the utmost” (*yòng xîn*, 用心), a way of moral cultivation, originating from *Mencius*, means one should fully understand and extend one’s innate goodness. Mencius holds that “He who exerting his heart to the utmost knows his nature. Knowing his nature, he knows the goodness inherent in the mind. Preserving his mind and nurturing his nature is the way to deal with his inherent morality” (Zhu, 2016, p. 310). Starting from the view that human nature is good, Mencius emphasizes that good human nature does not automatically constitute virtuous conduct and people need to continuously cultivate good human nature to develop virtuous conduct, eventually promoting the inherent “Four Hearts,” namely, commiseration, shame, deference, and sense of right and wrong (Zhu, 2016, p. 214). Zhu Xi comments, “The heart is the master of human beings, governing the nature of human beings, and containing the principle of all things in the world. Therefore, exerting one’s heart to the utmost enables people to grasp the laws, adapt to the environment, and manage affairs” (Zhu, 2016, p. 214). Confucius sets a good example of exerting his heart to the utmost by being determined to study at the age of 15 and followed his heart’s desire without going beyond what is right at the age of 70 (Zhu, 2016, p. 47–48). On the contrary, Xunzi believes that human nature is evil. If people are allowed to only follow desire for external things, which is something inherent in human nature, it will lead to conflicts, and society will fall into chaos. Moral conduct which is vital for maintaining order in society does not derive from human nature. Rather, it is acquired through deliberate efforts. Xunzi believes that although human nature is evil, as long as one can accumulate enough good to turn it into inner power, concentrate on accumulating knowledge and supplementing new information, then any man in the street can become a saint like Yu as long as he cultivates himself wholeheartedly (Wang, 2012, p. 385). All the above mentioned Confucian scholars are aimed at promoting the moral cultivation of discovering and nurturing the goodness inherent in the mind. The idea of “Exerting one’s heart to the utmost” has something in common with meditation training in mindfulness therapy. Meditation training is to guide the subject to activate the autonomic nervous system, stop all external activities of the mind, and achieve a state of self-forgetfulness in deep tranquility. Similarly, “Exerting one’s heart to the utmost” is to eliminate distractions and concentrate on extending one’s innate goodness, eventually realizing the moral qualities of benevolence, righteousness, rites and social norms, and wisdom and stay in Harmony with heaven. If one can exert his heart to the utmost toward fully cultivating his inherent kindness, he can consequently become a man of virtue.

Although the “Internal Harmony” belongs to the field of individual self-cultivation, it is not limited to the pure individual subjective psychological experience; rather, it requires the individuals to conform to the external social norms such as benevolence, righteousness, propriety, wisdom, and faith. “Internal Harmony” serves “External Harmony” and provides a strong internal driving force and internal support for “External Harmony,” which organically unifies individual freedom and community spirit, effectively overcoming the negative impact of extreme individualism, which neglects social responsibility, and collectivism, which ignores individual rights. Particularly in the context of the COVID-19 pandemic, healthcare workers suffer from the elevated risk of stress, burnout, trauma, and other negative mental health challenges as other ordinary people do (Søvold et al., 2021). However, still a large number of health workers go to the frontlines of the pandemic without hesitation because they hold the mission of group obligation in their hearts and shoulder the responsibility of saving lives. Probably that also accounts for how common people respond to social confinement measures imposed by governments in fighting against COVID-19 (Dos Santos et al., 2020). It is a community obligation that is responsible for their willingness to conscientiously cooperate with the government’s various epidemic prevention and control measures, such as keeping social distancing and staying home as much as possible.

## Conclusion

With the improvement of modern public health care, when the threat of physiological diseases to human health is relatively reduced, due to the accelerating pace of life and work as well as the increasing material desires among human beings, people suffer greater mental pressures accordingly, which is more likely to contribute to psychological conflicts, psychological imbalances, psychological breakdowns, and other psychological problems. In the context of this era, the pre-Qin Confucian thought of valuing Harmony, based on the trinity of achieving “Balanced Harmony” by following the golden mean, attaining “External Harmony” by benevolent people love others, and realizing “Internal Harmony” by means of seeking from the heart, which blend into one harmonious whole through internal and external efforts, constitute a well-knit integrated system. With rich mental health wisdom and superb psychological regulation methods, the concept of valuing Harmony is not only a precious psychological heritage that acts on the past, but also a precious psychological resource cast in reality, which has an important reference for maintaining people’s mental health in modern society worldwide and is surely worthy of further exploration.

## Author Contributions

YL has made substantial contributions to the collection of materials and drafting and translating of the work. HC has made great contributions to the conception of the work and the revision of the manuscript. Both authors listed have made a substantial, direct and intellectual contribution to the work, and approved it for publication.

## Conflict of Interest

The authors declare that the research was conducted in the absence of any commercial or financial relationships that could be construed as a potential conflict of interest.

## Publisher’s Note

All claims expressed in this article are solely those of the authors and do not necessarily represent those of their affiliated organizations, or those of the publisher, the editors and the reviewers. Any product that may be evaluated in this article, or claim that may be made by its manufacturer, is not guaranteed or endorsed by the publisher.
